# Sexual development in ADHD and internet pornography consumption

**DOI:** 10.3389/fpsyt.2023.1240222

**Published:** 2023-08-08

**Authors:** Michal Privara, Petr Bob

**Affiliations:** Center for Neuropsychiatric Research of Traumatic Stress, Department of Psychiatry and UHSL, First Faculty of Medicine, Department of Psychiatry, Faculty of Medicine Pilsen, Charles University, Prague, Czechia

**Keywords:** ADHD, pornography, compulsive behavior, hypersexuality, anxiety, depression

## Abstract

Development of sexual identity during adolescence is a major process of transition in individual life which in cases of ADHD specifically interacts with other ontogenetic, pathological and psychosocial conditions. According to recent findings growing consumption of internet pornography mainly in male ADHD population is closely related to compulsive sexual behavior and hypersexuality. Recent findings also indicate that consumption of internet pornography in ADHD individuals and other sexual activities may serve as a mood-altering “self-medication” which may help to cope with stressful events and decrease depression and anxiety. Taken together recent findings indicate that internet pornography consumption mainly in ADHD individuals is closely related to stressful experiences, anxiety, depression and identity problems in partnerships which significantly increase their vulnerability to the so-called “problematic pornography use” and other forms of addictive sexual behavior. From this developmental perspective “problematic pornography use” in ADHD individuals represents significant epidemiological problem which requires further research mainly with focus on clinical diagnostics and treatment.

## Introduction

Development of sexual identity is a major process of transition in individual life that is mainly established during adolescence, and it is related to various physiological, anatomical and behavioral changes leading to development of sexual activities and fantasies (1–4). Mainly, sexual maturation, various emotional changes, development of abstract thinking, sexual identity, self-consciousness and also anatomical changes related to rapid physical growth (5–7). This complex developmental process is related to various internal and external factors, including various ontogenetic, neuropathological, social and environmental conditions that may influence sexual functions and behavior (2, 5, 7–14).

One of these specific pathological influences that may negatively affect normal development of sexual functioning and behavior is Attention Deficit and Hyperactivity Disorder (ADHD) mainly because executive dysfunctions usually related to ADHD may cause various problems mainly related to impulsivity and hypersexuality, aggressive behavior and other problems which may negatively affect psychological development of sexual identity and sexual functioning (7, 13, 15). According to recent meta-analyzes ADHD manifests worldwide and affects at about 5.3% of child population and 2.5% of adults (5, 7, 13–18). The core symptoms of ADHD are inattention, hyperactivity and impulsivity and the disease is closely associated with various problems in daily performing and social functioning and adaption (19, 20) including difficulties in academic and professional life, and interpersonal relationships in comparison to individuals without ADHD (19–21).

Nevertheless, the diagnosis of ADHD as a developmental disorder according to the DSM-5 requires clear evidence that the symptoms may reduce the quality of life and everyday functioning mainly associated with a variety of risks factors (22–24). Usually individuals with ADHD manifest poor school performance and their professional success is often negatively impaired in comparison to individuals without ADHD (25, 26). In addition, there is evidence that ADHD also increases the risk of criminal behavior and various forms of rule violations (27, 28) as well as significant deficits in interpersonal relationships (5, 7, 29–33).

In this context, some studies also reported lower relational satisfaction, impaired quality of romantic relationships, higher rate of separations and divorces, increased risk of intimate partner violence and verbal violence (29, 34) and more maladaptive coping strategies in ADHD individuals (3, 4, 6, 35, 36). Additionally, people with ADHD are reported to be victims of sexual violence more frequently (37). Regarding a direct influence of ADHD on development of sexual functions recent findings suggest that no differences were found regarding the age of puberty onset in children with ADHD, and ADHD does not appear to be associated with delayed or impaired sexual maturation in comparison with children without ADHD (38–41).

## Attention deficit and hyperactivity disorder impulsivity and hypersexuality

Although ADHD likely has no direct influence on individual development and on sexual maturation in comparison to individuals without ADHD there is evidence that psychological development of sexual identity and functions is significantly affected by ADHD (7, 13, 15). In addition ADHD has frequent manifestations of comorbidities mainly mood disorders and anxiety disorders (15, 32, 39, 41, 42) and a frequent comorbid disorder related to ADHD is hypersexuality (7, 13, 32). Some studies also show positive associations between impulsivity and hypersexuality (1, 4, 42, 43) and inattentive symptoms also may be related to hypersexuality (32, 44, 45). In addition some findings suggest that sexual activities may serve as mood-altering “self-medication” process in some individuals with ADHD (7, 13, 32, 42, 46–48).

Basic mechanism for this “self-medication” might be reward deficiency that is typical of ADHD (49) which can be related to addictive, impulsive, compulsive and risky sexual behaviors (5, 7, 50–52). Other studies indicate that conduct problems during childhood and adolescence could also be a relevant factor of the relationship between ADHD and sexual risk behaviors (13, 53, 54). In addition conduct problems are often associated with increased impulsive behaviors (7, 13, 55–58).

In this context, some findings suggest that risky sexual behaviors in ADHD individuals represent a dysfunctional form of emotional regulation (7, 13, 59, 60), which may be also a relevant risk factor for sexual victimization (61–68). In addition, some research studies show that people with ADHD have increased tendencies to promiscuity and according to recent findings ADHD individuals have more sexual partners than people without ADHD (23, 69–72).

## Compulsive sexual behavior, problematic pornography use and ADHD

According to recent findings hypersexuality is frequently related to compulsive form of sexual behavior (CSB) usually characterized by unsuccessful effort to control excessive sexual behavior that may lead to various psychopathological symptoms and negatively affect close relationships and partnership, and significantly decrease quality of life (1, 3, 4, 6, 47, 61, 62, 66, 73–77). A highly prevalent form of compulsive sexual behavior in ADHD individuals is “problematic internet pornography use” (PPU) (6, 7, 13, 47, 59, 60, 76–79).

In this context, some studies indicate a strong association between ADHD and CSB (7, 13, 59, 60, 80, 81) and other findings show that ADHD represents a risk factor for problematic pornography use (PPU) as a manifestation of hypersexual phantasies (1, 3–6, 82). For example, a recent study examining the motivations behind Internet porn use found that men revealed much stronger motivations than women and those with more erotophilic tendencies were more motivated to use Internet pornography (6, 47).

As epidemiological findings indicate, PPU in ADHD population but also in the general population became a widespread problem for mental health due to unlimited access of pornographic material on the internet and the availability of the internet (6, 61, 62, 76, 80, 83–86) and recent evidence shows that a significant percentage of people (5–14%) who use pornography may encounter problematic pornography use (PPU) (6, 61, 62, 80, 83, 87, 88).

In the 11th edition of the International Classification of Diseases (ICD-11), PPU is characterized by persistent, excessive, or compulsive use of pornographic content and intensive participation in it, despite distress and adverse consequences (7, 13, 59, 60, 78, 80, 81, 87, 89–93). Nevertheless, some researchers consider the conceptualization of PPU as an impulse control disorder or a highly addictive form of sexual behavior (39, 94, 95).

Some studies also suggest that ADHD symptoms may represent significant predictive factor for PPU (5–7, 96–98) which is in agreement with other findings documenting that impulsivity, problematic sexual behaviors, CSB, hypersexuality are significantly related to PPU (1, 3–6, 84, 99). For example, Niazof et al. (7) reported a high rate of co-occurring ADHD in hypersexual people and found that nearly a quarter of 361 treatment-seeking hypersexual adult men were diagnosed with ADHD and that majority of them (96.4%) met diagnostic criteria for the inattentive subtype of ADHD. Other studies also indicate that PPU can be considered as a specific form of hypersexual behavior (1, 7, 42, 80, 83) and ADHD symptoms have moderate positive associations with hypersexuality in both men and women (5, 7, 61, 62, 80, 83). Other findings show that ADHD individuals report increased sexual desire, more masturbation frequency, less sexual satisfaction, and more sexual dysfunctions than the general population (6, 47, 61, 62, 80, 83).

In addition some findings suggest that hypersexuality and PPU might be related to anxiety due to difficulties in forming a secure attachment during individual psychological development (100–102). Due to this deficit people with anxious insecure attachment may be more vulnerable and tend to seek sexual relationships without appropriate emotional intimacy (6, 47, 77, 78, 103–105). These developmental attachment deficits seem be confirmed by findings that anxiety disorders represent significant comorbid conditions of ADHD (15, 106, 107) and these anxiety symptoms are related to pornography consumption (6, 108–114). In this context, other studies documenting PPU epidemiology indicate that PPU is mainly related to symptoms of anxiety and depression (41, 76, 89, 115–120), and also to obsessive–compulsive symptoms (6, 47, 121).

This severity of PPU as a mental health problem is documented by recent research which indicates that more than 80% of people seeking treatment for a compulsive sexual behavior disorder reported an inability to control their use of pornography, despite negative consequences (41, 78, 122–126). Typical negative consequences of PPU may lead to various psychological and physiological deficits and symptoms, as for example depressive symptoms, guilty feelings, anxiety symptoms, various negative consequences related to forming of sexual identity, body image problems, decreased ability to create relationships with potentional partners, emotional numbness, tendencies to social isolation, increased aggression and violent behavior, and may also negatively affect sexual functioning such as hypersexuality, erectile dysfunctions and also some sexual dysfunctions in women such as decreased sexual desire or anorgasmia (6, 78).

This increased interest to experience online pornography likely will increase susceptibility to PPU not just in ADHD individuals but also in general population and as some findings indicate PPU is associated with various sexual dysfunctions (127–132).

## Discussion

Recently mental health problems related to pornography use became epidemiologically significant mainly because of severe increase in online pornography consumption by pre-adolescent children and adolescents (9–12, 76, 78), which can have serious future adverse consequences [(47, 89, 118, 128, 133, 134); Gola et al., 2016].

From this perspective further research regarding PPU and its clinical diagnostics and treatment represent significant epidemiological problem. Although the Compulsive Sexual Behavior Disorder (CSBD) is included in the International Statistical Classification of Diseases and Related Health Problems (ICD-11) (135), there is no official diagnosis for the problematic pornography use (PPU) (136). Nevertheless, since PPU is often considered a manifestation of CSBD, the same diagnostic guidelines can be applied (136).

Currently the evidence-based treatment methods of PPU, other forms of problematic online sexual behavior and CSBD in ADHD and other psychiatric patients (126, 137–143), mainly include cognitive behavioral therapy (CBT), acceptance and commitment therapy (ACT), interview techniques motivational and mindfulness based approaches (126, 137–143).

In this context of the treatment of ADHD individuals it is important to take into account that their PPU related problems may be related to their “reward deficiency” and “increased sensitivity” (49, 54, 71, 76, 144, 145). Mainly because there is evidence that the prefrontal cortex (PFC) is overactive in people with ADHD (8, 15, 146) that is related to attention deficits, which may cause a self-regulatory attempts (called as “self-medication” behavior) manifesting as increased needs to experience sexual desire (6, 32, 76, 78, 147). From these perspectives PPU and other addictive forms of sexual behavior in ADHD individuals represent significant epidemiological problem which requires further clinical research mainly focused on underlying psychophysiological mechanisms, clinical diagnostics and treatment options.

## Data availability statement

The original contributions presented in the study are included in the article/supplementary material, further inquiries can be directed to the corresponding author.

## Author contributions

MP and PB did the research and wrote the manuscript. All authors contributed to the article and approved the submitted version.

## Funding

This research was supported by Charles University project Cooperatio SVV.

## Conflict of interest

The authors declare that the research was conducted in the absence of any commercial or financial relationships that could be construed as a potential conflict of interest.

## Publisher’s note

All claims expressed in this article are solely those of the authors and do not necessarily represent those of their affiliated organizations, or those of the publisher, the editors and the reviewers. Any product that may be evaluated in this article, or claim that may be made by its manufacturer, is not guaranteed or endorsed by the publisher.
